# Supplementary material for: A neuromechanistic model for rhythmic beat generation
Source: PLoS Comput Biol. 2019 May 9;15(5):e1006450. doi: 10.1371/journal.pcbi.1006450 (PMC6508617; doi:10.1371/journal.pcbi.1006450)
Supplement: S1 Appendix — Full description of the model, model parameter and numerical simulation schemes. (PDF) [file pcbi.1006450.s001.pdf]

Supporting information

S1 Appendix. The model for the BG consists of a set of biophysical conductance-based equations which we call the I_{NaP} model that incorporate a low-threshold calcium current I_{CaT}, a sag current, I_h, a persistent sodium current I_{NaP} and a leak current, I_L. The current balance equations are given by

$$\begin{aligned} C\frac{dV}{dt} &= I_{\text{bias}} + I_{int} - g_L[V - E_L] - g_{CaT}m_\infty(V)h[V - E_{Ca}] \\ &\qquad - g_h r[V - E_h] - g_{NaP}a_\infty(v)[v - E_{Na}] && (1) \\ \frac{dh}{dt} &= \frac{h_\infty(V) - h}{\tau_h(V)} && (2) \\ \frac{dr}{dt} &= \frac{r_\infty(V) - r}{\tau_r(V)} && (3) \end{aligned}$$

The term I_{bias} refers to a drive whose value determines whether the isolated BG can oscillate and if so, at which frequency. We set $I_{int} = -33\mu\text{A/cm}^2$ so that if $I_{\text{bias}} = 0\mu\text{A/cm}^2$ there are no oscillations. The parameters $g_{CaT}, g_h, g_{NaP}, g_L$ and E_{Ca}, E_h, E_{Na}, E_L refer to conductances and reversal potentials for the calcium, sag, sodium and leak currents, respectively. The functions $x_\infty(V) = 1/(1 + \exp(-(V - v_x)/k_x))$ for $x = m, a, r, h$ are each sigmoidal functions with half-activation voltages v_x and accompanying slopes k_x. The time constants are $\tau_h(V) = \tau_L/(1 + \exp((V - v_h)/k_h)) + \tau_R(1 + \exp(-(V - v_h)/k_h))$ and $\tau_r(V) = \tau_{r_{max}}/\cosh((V - v_{r_\tau})/(2k_{r_\tau}))$. The T-current is considered to have instantaneous activation, modeled by $m_\infty(V)$ and a slow inactivation, governed by the h variable. The sag current simply has a slow activation variable r. The persistent sodium current has just instantaneous activation given by $a_\infty(V)$. The parameter values are as follows: $C = 1$, $g_{CaT} = 11$, $g_h = 1$, $g_{NaP} = 0.1$, $g_L = 1.6$, $E_{Ca} = 50$, $E_h = -30$, $E_{Na} = 50$, $E_L = -70$, $v_m = -40$, $k_m = 6.5$, $v_a = -67$, $k_a = 1$, $v_r = -70$, $k_r = 12$, $v_h = -60$, $k_h = 6$, $v_{r_\tau} = -75$, $k_{r_\tau} = 8$, $\tau_L = 30$, $\tau_R = 5$, $\tau_{r_{max}} = 850$ (where capacitance is in units of μF/cm^2, conductances are in mS/cm^2, time constants are in ms and all others parameter units are mV):.

The equations for the S neuron are

$$\begin{aligned} C_S\frac{dV_S}{dt} &= I^S_{bias} + g_{stim}I_{stim}(t) - g_{L_S}[V_S - E_L] - g_{CaT_S}m_\infty(V_S)h_S[V_S - E_{Ca}] && (4) \\ \frac{dh_S}{dt} &= \frac{h_\infty(V_S) - h_S}{\tau_h(V_S)} && (5) \\ & && (6) \end{aligned}$$

The term $I_{stim}(t)$ is the periodic current provided from the stimulus. During each cycle, it is positive for 25 ms and 0 otherwise. It is taken to be large enough to ensure that S fires within 5 ms of sound onset times. The m_∞, h_∞ and τ_h functions are as above, the parameter values are the same unless otherwise stated: $I^S_{bias} = -14$, $g_{stim} = 6$, $g^S_{CaT} = 10$ (units as above).

The γ counters are constructed as follows. Solve $x' = -x/\tau_x$ with $x(0) = 2$ until it reaches 1 at $t = t_g$ and is reset to 2; $x(t_g^-) = 1$ reset to $x(t_g^+) = 2$. The counters keep track of the number of resets. We chose $\tau_x = 40$ ms which yields an inter-spike interval of 27.73 ms (frequency of 36.06 Hz). Heterogeneity between the BG and S counters of roughly 10% was introduced to the IOI_S by varying τ_x. In the case of stochasticity, Ornstein-Uhlenbeck noise was added to the x variable, with a timescale of 5 ms and Gaussian white noise with mean 0 and standard deviation 0.005. All numerical simulations were carried out in MATLAB.
